# Wide-Spectrum Microscope with a Long Working Distance Aspherical Objective Based on Obscuration Constraint

**DOI:** 10.3390/s16111886

**Published:** 2016-11-09

**Authors:** Weibo Wang, Chao Wang, Jian Liu, Jiubin Tan

**Affiliations:** 1 Institute of Ultra-Precision Optoelectronic Instrument Engineering, Harbin Institute of Technology, Harbin 150001, China; liujian@hit.edu.cn (J.L.); jbtan@hit.edu.cn (J.T.); 2 Department of Engineering Science, University of Oxford, ParksRoad, Oxford OX1 3PJ, UK

**Keywords:** wide-spectrum microscope, aspherical Schwarzschild objective, low obscuration, long working distance

## Abstract

We present an approach for an initial configuration design based on obscuration constraint and on-axis Taylor series expansion to realize the design of long working distance microscope (numerical aperture (NA) = 0.13 and working distance (WD) = 525 mm) with a low obscuration aspherical Schwarzschild objective in wide-spectrum imaging (λ = 400–900 nm). Experiments of the testing on the resolution target and inspection on United States Air Force (USAF) resolution chart and a line charge-coupled device (CCD) (pixel size of 14 μm × 56 μm) with different wavelength light sources (λ = 480 nm, 550 nm, 660 nm, 850 nm) were implemented to verify the validity of the proposed method.

## 1. Introduction

The typical working distance (WD) of an objective depends on the numerical aperture (NA) and ranges from tens or hundreds of micrometers to approximately 30 mm. The design of an objective with a working distance of hundreds of millimeters and a relatively larger NA is quite difficult, because the aberration will grow proportionally with respect to the size of pupil apertures. However, such an optical microscope objective is somewhat irreplaceable when high lateral imaging resolution and sensitive axial position detection are concurrently needed. For the large areas measurements by sub-aperture stitching, such as imaging a multiwindow wide-spectrum detector, the z defocusing should be controlled to within 1 μm. When measuring over large areas using subaperture stitching, such as imaging a multiwindow wide-spectrum detector, a long working distance to satisfy mechanical clearance requirements and relatively high NA objectives to inspect structures with suitable lateral resolution are needed. The design of microscopes with an objective (WD ≥ 500 mm, NA ≈ 0.15, λ = 400–900 nm) is very difficult. Chromatism control is the most problematic design constraint, which is proportional to aperture size, focal length, and wavelength range. Additionally, a long WD usually results in a complex design and high cost. Therefore, it may not be a good idea to try to put the design into lenses.

Mirror technology is a known chromatic free solution, such as Schwarzschild mirror, which has been successfully applied in X-ray imaging [1,2], ultraviolet imaging under cryogenic conditions [3], and infrared sources [4]. However, such coaxial reflective systems have the presence of central beam obscuration, which significantly reduces the illumination efficiency and degrades the image contrast [5]. A high obscuration ratio (OR) could result in significant degradation of contrast and ultimately results in low stitching accuracy, since the initial contrast of some structures, such as charge-coupled device (CCD) pixels, is only 0.2–0.3. Therefore, the main task of the design is to suppress the OR and enlarge the WD.

Aspherical mirrors provide an approach to get a low OR, which makes use of the Abbe sine and constant optical path conditions [6] or fits an equation to the numerical data representing the primary and secondary mirror surfaces of the microscope [7]. However, the calculation is complex and the control of the OR is not yet included in the analytical design procedures [8,9,10].

In this paper, we present an approach for the initial configuration design of an aspherical Schwarzschild objective with a low OR, a large WD, and a source wavelength range of 400–900 nm. The discussion focuses on the design and application of aspheric reflection objective based on obscuration constraint.

## 2. Methods

**Figure 1:**
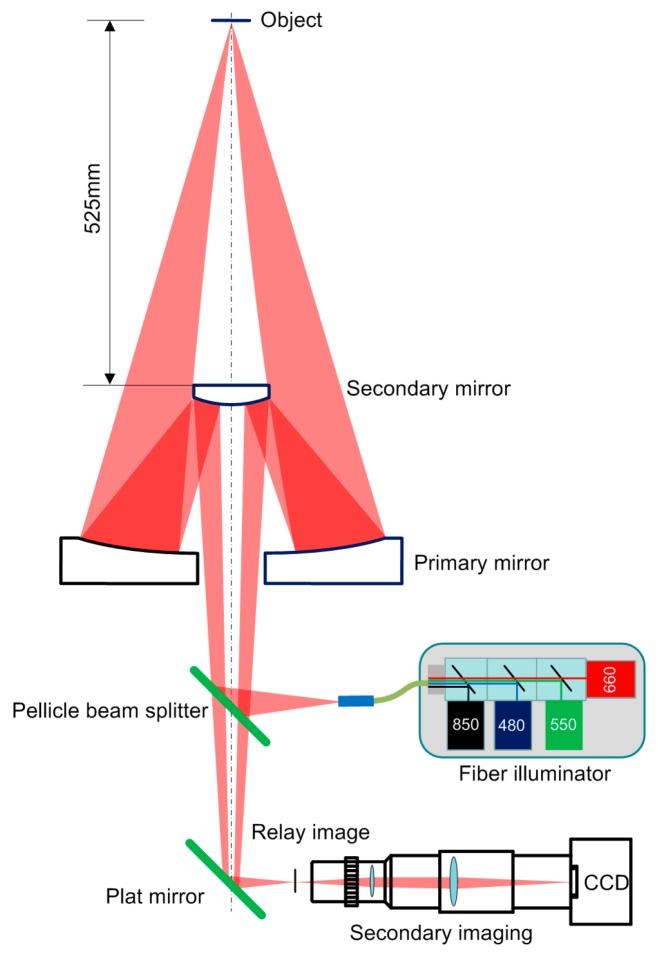
Scheme of a microscope with a Schwarzschild objective.

**Figure 2:**
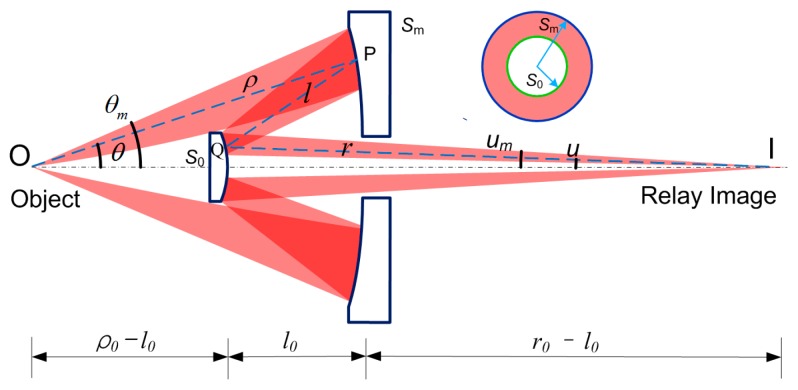
Parametric diagram of the Schwarzschild objective.

The scheme of a wide-spectrum microscope with a long working distance aspherical Schwarzschild objective (WD = 525 mm, NA = 0.13, magnification m = 6.5×, λ = 400–900 nm) is shown in Figure 1. The Schwarzschild objective consists of primary and secondary aspheric mirrors, as shown in Figure 2.

The primary and secondary mirrors are denoted by polar coordinates (*ρ*, *θ*) and (*r*, *u*) with origin points at O and I, respectively. The distance PQ is *l*. The two mirror vertexes are at *ρ* = *ρ*_0_ and *r* = *r*_0_. The mirror vertex distance is *l*_0_. The maxima of *θ* and *u* are *θ*_m_ and *u*_m_, respectively. The areas of secondary and primary mirrors within the maximum aperture angles *θ*_m_ and *u*_m_ are defined as *S*_0_ and *S*_m_. It can be seen clearly that the size and position of the coaxial secondary mirror affects the beam obscuration.

The obscuration ratio reflects the level of beam shading and how the light is blocked during illumination and imaging. For the coaxial reflective systems with central beam obscuration, the pupil function can be expressed as:

(1) $$P(r)=\{\begin{array}{cc} 1, & \varepsilon a\leq r\leq a\\ 0, & r>a\;\mathrm{or}\;r<\varepsilon a \end{array}$$

where *a* is the outer diameter of the annular pupil, and *ε* is the ratio of the inner and outer diameters.

The amplitude distribution function on the focal plane can be written as:

(2) $$U(r^{\prime })=\frac{i\pi a^{2}}{\lambda f}\exp(-ikf)\exp\left(-\frac{i\pi r^{2}}{\lambda f}\right)\cdot \left[\frac{2J_{1}\left(\frac{2\pi r^{\prime }a}{\lambda f}\right)}{\frac{2\pi r^{\prime }a}{\lambda f}}-\varepsilon^{2}\frac{2J_{1}\left(\varepsilon \frac{2\pi r^{\prime }a}{\lambda f}\right)}{\varepsilon \frac{2\pi r^{\prime }a}{\lambda f}}\right].$$

It can be seen clearly that the presence of a central beam obscuration affects the amplitude distribution directly, which significantly reduces the illumination efficiency and degrades the image contrast.

OR can be modeled as a constraint parameter (*S*_0_/*S*_m_) to control beam obscuration. From the definition of *S*_0_/*S*_m_ and paraxial condition, the following expression can be obtained:

(3) $$S_{0}/S_{m}=\frac{{\left(r_{0}\tan u_{m}\right)}^{2}\pi }{{\left[(\rho_{0}-l_{0})\tan\theta_{m}\right]}^{2}\pi }$$

where *θ* is the angle between the input rays and the optical axis, and *u* is the angle between the output rays and the optical axis.

Because *θ* and *u* are always quite small, we can obtain:

(4) $$\frac{\tan u}{\tan\theta }\approx \frac{\sin u}{\sin\theta }.$$

Furthermore, by the Abbe sine and constant optical path conditions, sinθ=msinu (m is the system magnification), the OR can be expressed as

(5) $$S_{0}/S_{m}=\frac{{\left(r_{0}\tan u_{m}\right)}^{2}\pi }{{\left[(\rho_{0}-l_{0})\tan\theta_{m}\right]}^{2}\pi }={\left(\frac{r_{0}}{m(\rho_{0}-l_{0})}\right)}^{2}.$$

As illustrated in Figure 2, $b=\rho_{0}-l_{0}$ is the sum of WD and the secondary mirror thickness (which is always neglected in the design procedures). Therefore, the OR is determined by the working distance, system magnification, and the distance between the secondary mirror and the focal plane.

The total length of the Schwarzschild objective can be written as:

(6) $$L=\rho_{0}-l_{0}+r_{0}.$$

By taking Equation (5) into Equation (6), we can obtain:

(7) $$L=(m+1)(\rho_{0}-l_{0})\sqrt{\mathrm{OR}}.$$

It can be seen clearly that the OR should be reduced to get a long working distance and a compact structure. Therefore, the control of the OR should be included in the analytical design procedures. In practice, *S*_0_/*S*_m_, *m*, and *ρ*_0_−*l*_0_ should be determined according to the requirements firstly. Then, *r*_0_ and *ρ*_0_ can be optimized based on fabrication experiences. The final parameters can be obtained by computer-aided design and iterative optimization.

**Figure 3:**
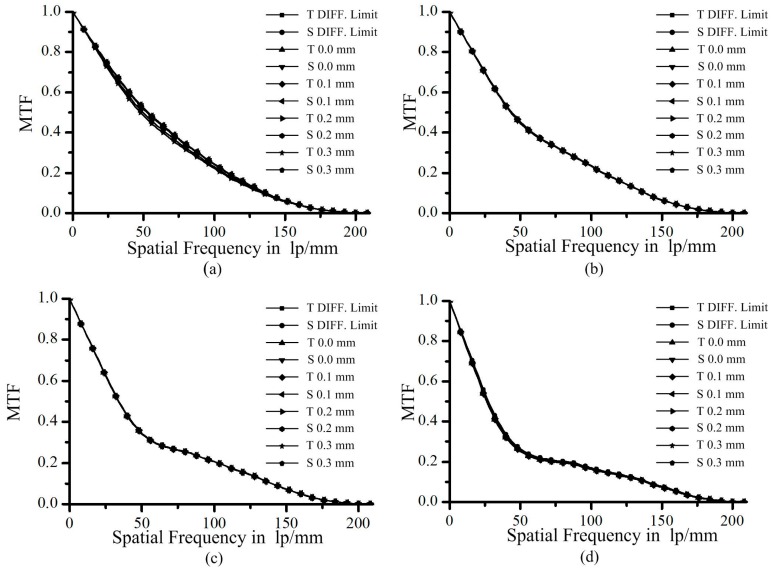
Modulation transfer functions (MTF) of reflective objectives with different obscurations. (**a**) OR = 5%; (**b**) OR = 10%; (**c**) OR = 20%; (**d**) OR = 30%.

For the assumed values, *ρ*_0_ = 150 mm, *l*_0_ = 50 mm, *m* = 4, OR is 5%, 10%, 20%, and 30% respectively, the field of view (FOV) is 0, 0.1 mm, 0.2 mm, and 0.3 mm, respectively, the NA of objective is 0.2, and we can obtain the modulation transfer functions (MTF) of reflective objectives with different obscurations, as shown in Figure 3.

It can be seen clearly that the MTF amplitude of curve decreases in the low and middle frequencies with the increase in OR. Compared with the MTF of Figure 3a, the MTF amplitude of Figure 3c,d is lower before the point of 50 lp/mm and slightly enhanced after this point. From experience, the low amplitude of the MTF at the low and middle frequencies will result in a blurred image, while an enhanced high-frequency MTF will result in a sharper edge. Compared with the CCD being stitched here, the pixel and the microcircuit wire contrast is 0.2–0.3. Hence, the central parts of the MTF of Figure 3c,d are too weak to allow inspection of the pixels and wires. The enhancement on the high-frequency MTF is of limited contribution to the requirements here. Moreover, all the curves overlap with their diffraction limit and cut off at the same frequencies. The validity of the proposed method based on obscuration constraint can be verified by the above simulations.

After *ρ*_0_, *l*_0_, and *r*_0_ are selected by the method mentioned above, a theoretical approach for the aspheric surface parameters, such as the curvature, conic constant, and high-order coefficients has been modeled using an on-axis Taylor series expansion [11]. Therefore, the parameters of a low obscuration aspherical Schwarzschild objective can be obtained. The radius of curvature *R*_1_ and the conic constant *k*_1_ of primary aspheric mirror can be determined as follows:

(8) $$\left\{ \begin{array}{l} OR={\left[\frac{r_{0}}{m(\rho_{0}-l_{0})}\right]}^{2}\\ R_{1}=\frac{2ml_{0}\rho_{0}}{ml_{0}+m\rho_{0}-r_{0}}\\ \begin{array}{ll} k_{1}= & -\frac{{\left(R_{1}-\rho_{0}\right)}^{2}}{\rho_{0}^{2}}+\frac{r_{0}R_{1}^{3}(m+1)}{m^{2}\rho_{0}^{3}(\rho_{0}+r_{0})}[\frac{\kappa^{3}{\left(m+1\right)}^{3}{\left(m-1\right)}^{2}}{4m{\left(m\kappa -1\right)}^{2}{\left(m-\kappa \right)}^{2}}\\ & -\frac{\kappa (m+1)(m-1)}{2(m\kappa -1)(m-\kappa )}+\frac{\kappa {\left(\kappa +1\right)}^{2}(m-1)(2-\alpha -\beta )}{4(m\kappa -1)(m-\kappa )}\\ & -\frac{\left(2-\alpha -\beta )(\kappa +1)(\alpha +m\beta -1\right)}{2m}+\frac{\left(m+1)(\alpha +m\beta \right)}{2m^{2}}\\ & -\frac{1}{m}] \end{array} \end{array}\right.$$

where $\kappa =\left(\rho_{0}+r_{0}\right)/l_{0}$, α=mκ/(Mκ−1), β=m/(m−κ), *M* = 1/*m*.

Further, the expressions for the vertex radius of *R*_2_ and the conic constant *k*_2_ of the secondary mirror can also be determined in a similar way:

(9) $$\left\{ \begin{array}{l} OR={\left[\frac{r_{0}}{m(\rho_{0}-l_{0})}\right]}^{2}\\ R_{2}=\frac{2l_{0}r_{0}}{r_{0}+l_{0}-m\rho_{0}}\\ \begin{array}{ll} k_{2}= & -\frac{{\left(R_{2}-r_{0}\right)}^{2}}{r_{0}^{2}}+\frac{\rho_{0}R_{2}^{3}(M+1)}{M^{2}r_{0}^{3}(r_{0}+\rho_{0})}[\frac{\kappa^{3}{\left(M+1\right)}^{3}{\left(M-1\right)}^{2}}{4M{\left(M\kappa -1\right)}^{2}{\left(M-\kappa \right)}^{2}}\\ & -\frac{\kappa (M+1)(M-1)}{2(M\kappa -1)(M-\kappa )}+\frac{\kappa {\left(\kappa +1\right)}^{2}(M-1)(2-\alpha^{\prime }-\beta^{\prime })}{4(M\kappa -1)(M-\kappa )}\\ & -\frac{\left(2-\alpha^{\prime }-\beta^{\prime })(\kappa +1)(\alpha^{\prime }+M\beta^{\prime }-1\right)}{2M}+\frac{\left(M+1)(\alpha^{\prime }+M\beta^{\prime }\right)}{2M^{2}}\\ & -\frac{1}{M}] \end{array} \end{array}\right.$$

where $\alpha^{\prime }=M\kappa /\left(M\kappa -1\right)$, β=M/(M−κ).

## 3. Experiment

For the target values (*S*_0_/*S*_m_ = 4%, NA = 0.13, *m* = 6.5, FOV ≤ 0.3 mm, and WD = 525 mm), $\rho_{0}-l_{0}=550\;\mathrm{mm}$, $l_{0}=190\;\mathrm{mm}$, and $r_{0}=715\;\mathrm{mm}$. The theoretical results are obtained by the proposed method based on obscuration constraint and on-axis Taylor series expansions and then optimized by Zemax. The primary and secondary mirrors are made of fused silica and coated with aluminum for high reflectivity in the spectral range from 400 to 900 nm, and an extra antioxidant coating for durable usage is finally added. The surface errors of the primary and secondary mirrors are about λ/10PV (λ = 632.8 nm).

**Figure 4:**
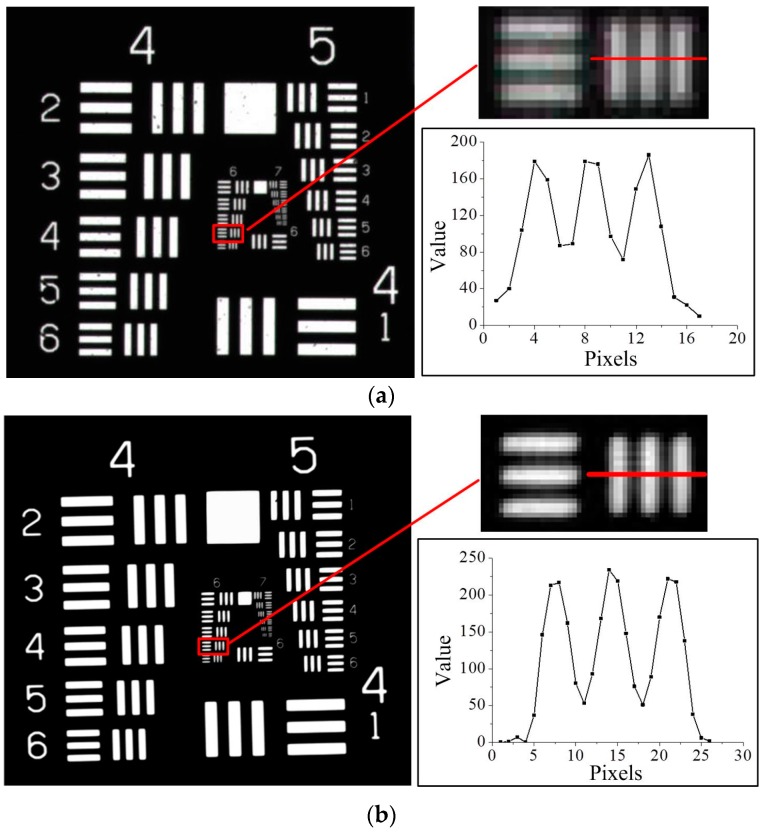
Raw image tests on the 1951 United States Air Force (USAF) resolution target (**a**) with the Olympus objective lens, PLN 4X; (**b**) with the developed objective.

For comparison, A standard 1951 United States Air Force (USAF) resolution chart is tested by a commercial microscope (NOVEL OPTICS, Ningbo, China, NMM-800) with an objective lens (Olympus, Tokyo, Japan, PLN 4X, NA = 0.1, WD = 18.5 mm) and the designed long working distance aspherical Schwarzschild objective (NA = 0.13, WD = 525 mm), respectively, as shown in Figure 4. These two objectives have similar NA. However, the WD of the latter is more than 28 times that of the former. For the latter, the white LED back illuminator (HFL-50-50-W, Dongguan CST, Dongguan, China) is placed behind the USAF target for contrast testing, and the image is recorded at the relay image plane. It can be seen clearly that the designed objective has a similar resolution with the Olympus objective lens, and the pattern (102 lp/mm) is resolved with high contrast.

**Figure 5:**
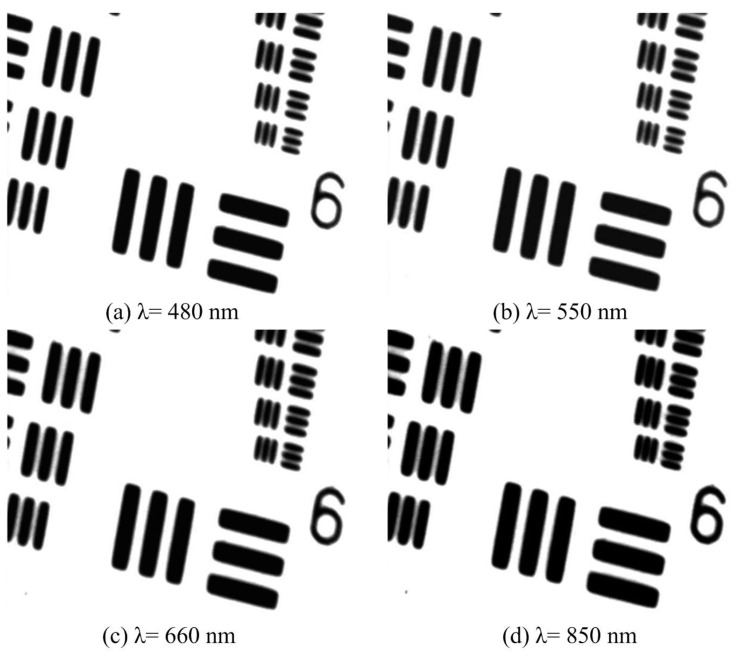
Raw image tests on the 1951 United States Air Force (USAF) resolution target with different wavelength light sources.

The secondary lens was used to provide a larger magnification and a larger range for fine positioning, and to reduce eye fatigue during lengthy observations. The 1951 USAF resolution target is also used to aid and test the long working distance microscope with λ = 480 nm, 550 nm, 660 nm, 850 nm and the secondary lens, as shown in Figure 1. The 7th pattern in the 6th group (228 lp/mm) was resolved with high contrast for different wavelength light sources, as shown in Figure 5. A clear image can still be obtained after the magnification of the secondary lens with high resolution and minor chromatism.

**Figure 6:**
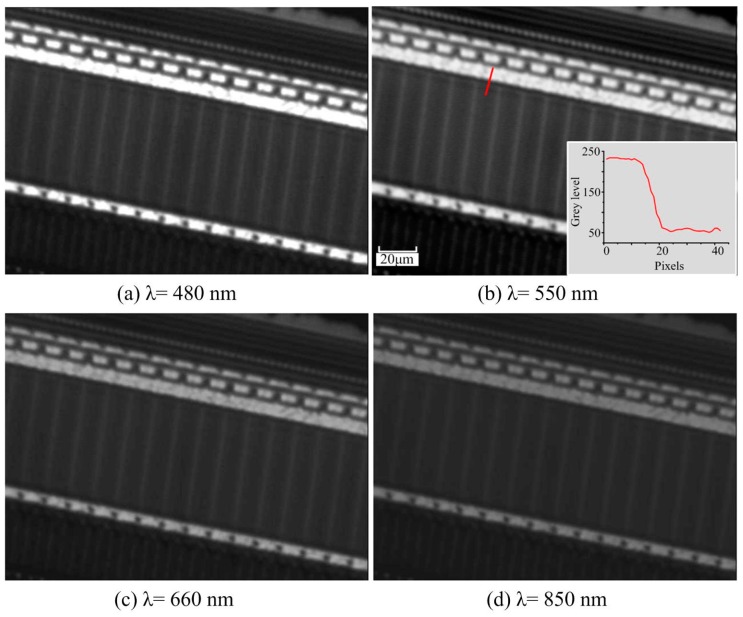
Inspections of a line charge-coupled device (CCD) by the developed long working distance (WD) microscope with different wavelength light illuminators.

Furthermore, Figure 6 gives the inspection results on a line CCD (LC100, Thorlabs, Newton, MA, USA) with a pixel size of 14 μm × 56 μm by the developed long WD microscope with a fiber illuminator (λ = 480 nm, 550 nm, 660 nm, 850 nm). The 1951 USAF resolution target and the line CCD are not used to test the limit of resolution but to investigate the difficulty of stitching inspection due to the weak pixel contrast, the microscale of the circuit wires, and the large WD. The size of the line CCD was chosen according to the requirements of the project that the long working distance microscope objective is developed for. The image dimension is 631 × 472 Pixels. The pixels and microcircuits are both clearly identified with different wavelength light sources, as are the small node grids.

## 4. Conclusions

The discussions on the design and application of a wide-spectrum microscope (λ = 400–900 nm, NA = 0.13 and WD = 525 mm) with a long working distance aspheric Schwarzschild objective constrained by obscuration have been herein presented. With the approach for the initial configuration design based on obscuration constraint and on-axis Taylor series expansion, the developed objective has a similar resolution but 20 times working distance compared with a commercial objective with a similar NA. Therefore, the design of long working distance microscope for some non-standard uses can benefit from the proposed method of an initial configuration based on obscuration constraint and on-axis Taylor series expansion. For designs of objective with high NA, the proposed approach can be applied and coupled with commercial software for global optimization to improve off-axis aberrations.
